# Detection and Genomic Characterisation of *Clostridioides difficile* from Spinach Fields

**DOI:** 10.3390/pathogens11111310

**Published:** 2022-11-08

**Authors:** Pilar Marcos, Paul Whyte, Catherine Burgess, Daniel Ekhlas, Declan Bolton

**Affiliations:** 1 Teagasc Food Research Centre, Ashtown, D15 DY05 Dublin, Ireland; 2 School of Veterinary Medicine, University College Dublin, Belfield, D04 V1W8 Dublin, Ireland

**Keywords:** *Clostridioides difficile*, spinach, soil, prevalence, WGS, antibiotic resistance, ribotype

## Abstract

Despite an increased incidence of *Clostridioides difficile* infections, data on the reservoirs and dissemination routes of this bacterium are limited. This study examined the prevalence and characteristics of *C. difficile* isolates in spinach fields. *C. difficile* was detected in 2/60 (3.3%) of spinach and 6/60 (10%) of soil samples using culture-based techniques. Whole genome sequencing (WGS) analysis identified the spinach isolates as belonging to the hypervirulent clade 5, sequence type (ST) 11, ribotypes (RT) 078 and 126 and carried the genes encoding toxins A, B and CDT. The soil isolates belonged to clade 1 with different toxigenic ST/RT (ST19/RT614, ST12/RT003, ST46/RT087, ST16/RT050, ST49/RT014/0) strains and one non-toxigenic ST79/RT511 strain. Antimicrobial resistance to erythromycin (one spinach isolate), rifampicin (two soil isolates), clindamycin (one soil isolate), both moxifloxacin and rifampicin (one soil isolate), and multi-drug resistance to erythromycin, vancomycin and rifampicin (two soil isolates) were observed using the E test, although a broader range of resistance genes were detected using WGS. Although the sample size was limited, our results demonstrate the presence of *C. difficile* in horticulture and provide further evidence that there are multiple sources and dissemination routes for these bacteria.

## 1. Introduction

*Clostridioides difficile* is an enteric spore-forming and toxigenic pathogen that was historically classified as a hospital-acquired infection [1]. Common symptoms range from watery non-bloody diarrhoea with abdominal pain to life-threatening fulminant colitis [2]. However, the steady increase in community acquired *C. difficile* infection(s) (CDI) in recent years has motivated investigative research on other sources and dissemination routes. As a result, specific *C. difficile* ribotypes, such as RT078, are now being classified as community-acquired [3,4].

Virulence in *C. difficile* is mainly determined by the production of toxin A (*tcdA*) and B (*tcdB*), which alter the host’s gut by causing damage to the epithelial barrier, leading to the translocation of commensal bacteria and cell death [5,6,7]. In addition, accessory genes *tcdR* and *tcdC* are part of the pathogenicity locus (PaLoc) and play a key role in regulating toxin production [8,9]. Apart from toxins A and B, it is estimated that up to 30% of *C. difficile* strains can produce the transferase *C. difficile* binary toxin (CDT), which belongs to the binary toxin family and is generally associated with hypervirulent strains [10,11,12].

Epidemiological investigations of *C. difficile* cases often use ribotyping that differentiates strains based on polymorphisms in the 16S-23S rRNA intergenic spacer region [13]. Certain ribotypes are more often linked with a higher occurrence and severity of disease in humans and some of these have been isolated from the food chain. Multi-drug resistant RT027, for example, is often isolated from clinical samples and is frequently associated with more severe illnesses and has been isolated from different food animals [14,15,16]. Moreover, RT014/020 and RT001/072 are confirmed endemic ribotypes in Europe [17].

In addition to its toxigenic virulence, antimicrobial resistance among the *C. difficile* strains is common worldwide, driven by the increased use of antibiotics as a treatment therapy for CDI [18]. For example, the emergence of *C. difficile* RT027 and its high resistance to fluoroquinolones is linked to the extensive use of these antibiotics in human cases [19]. Indeed, clinical isolates are often resistant to clindamycin, cephalosporins and penicillins, in addition to fluoroquinolones [20], with emergent strains also showing resistance to several antibiotics simultaneously [21].

While it is known that the bacterium colonizes the gut after transmission to a new host, the primary habitat of *C. difficile* is soil [22,23]. Thus, *C. difficile* spores have been frequently reported in soil on farms, in gardens and in forests [24,25,26]. Its ability to form spores facilitates survival and dissemination of this organism via organic fertilizers, wind and water. Thus, crops including vegetables are inevitably contaminated, resulting in a potential risk of infection for consumers [27,28,29,30]. Several studies have reported the detection of *C. difficile* in vegetables at retail [31,32,33], including salad leaves [24,34] and ready-to-eat (RTE) salads [31,35,36]. The *C. difficile* ribotypes identified in these studies include RT056, which has been associated with human illness [37]. However, further information is required to facilitate a better understanding of the role of vegetables in human CDI. Moreover, additional data on the genomic profile of the isolates from such sources would add to our knowledge of this emergent pathogen.

The objectives of this study were to (i) determine the prevalence of *C. difficile* in spinach and soil samples from fields where spinach was grown, (ii) characterise the *C. difficile* isolates originating from spinach and associated soil to determine their virulence and antimicrobial resistance using laboratory-based techniques and WGS, and (iii) compare the ribotypes and strains detected to those previously reported to establish if they are limited to the vegetable production environment or are part of the growing collection of *C. difficile* isolated from environmental, animal, food and/or human sources.

## 2. Materials and Methods

### 2.1. Sample Collection

**Figure 1:**
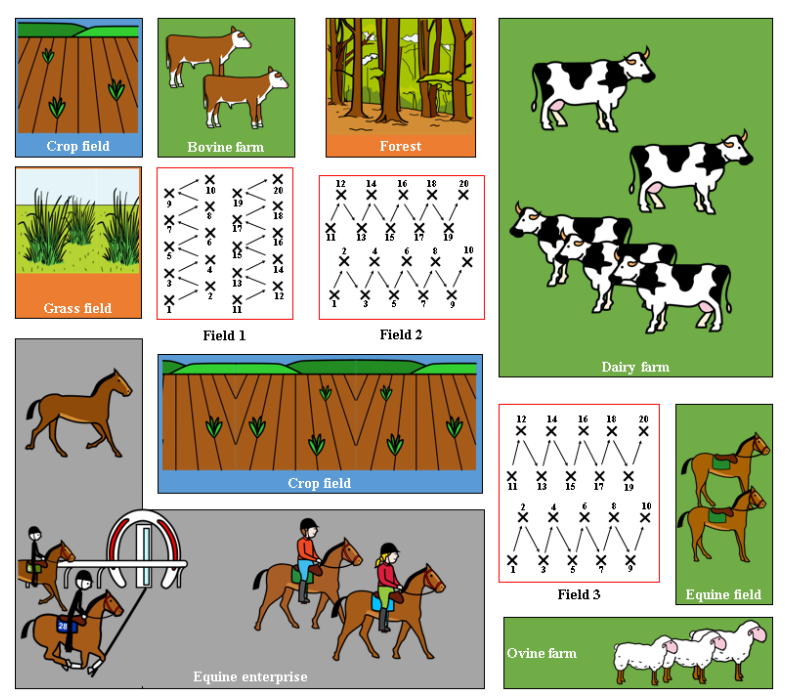
Layout of the 3 spinach fields used in this study and their surroundings. The sampling technique used to cover most of the area is detailed in each field.

Soil (n = 60) and spinach (n = 60) samples were obtained from three different spinach fields in the same area in Co. Meath (north-east Ireland). Field 1 and Field 2 were adjacent to each other, while Field 3 was bordered by a sheep farm on one side and a dairy farm on the other (Figure 1). In each field, 20 soil and 20 spinach samples were collected in 250 mL Ramboldi sterile containers (Sparks Lab Supplies, Rathcoole, Dublin, Ireland) using sterile sampling scoops (VWR, Ballycoolin, Dublin, Ireland). Soil samples were collected from directly under the spinach plants tested. In addition, sampling was carried out in a zig-zag pattern to cover the entirety of the field (Figure 1). Samples were then transported in a cool box (at approximately 4 °C) to the laboratory for testing within 24 h. None of the fields had been amended with slurry in the 12 months prior to the sampling date.

### 2.2. C. difficile Isolation and Confirmation

Soil and spinach samples were tested for *C. difficile* by adding 90 mL of maximum recovery diluent (MRD) to 10 g of each sample in a stomacher bag and blending for 1 min in a Star Blender LB 400 Stomacher (VWR, Lutterworth, Leicestershire, UK). To ensure the selection of *C. difficile* spores, vegetative cells were eliminated by heating at 60 °C for 25 min. To encourage spore germination, 10 mL of the heat-treated mixture was added to 90 mL of brain heart infusion broth (BHI) (Oxoid, Basingstoke, Hampshire, UK (CM1135B)) with 0.1% sodium taurocholate (Sigma-Aldrich, Gillingham, UK (86339-25G)) and selective *C. difficile* supplements (containing 8 mg/L of cefoxitin and 250 mg/L of D-cycloserine) (Oxoid, Basingstoke, Hampshire, UK (SR0096E)). Samples were incubated at 37 °C for 3–5 days under anaerobic conditions in an A35 Anaerobic workstation (Don Whitley, Victoria Works, West Yorkshire, UK) [38].

Exactly 10 µL of the broth culture was then streaked onto *C. difficile* moxalactam norfloxacin agar (Oxoid, Basingstoke, Hampshire, UK (CM0601B)) supplemented with 0.1% sodium taurocholate (Sigma-Aldrich, Gillingham, UK (86339-25G)) (CDMN-T), 32 mg/L moxalactam, 12 mg/L norfloxacin, 500 mg/L cysteine hydrochloride (Oxoid, Basingstoke, Hampshire, UK (SR0173)) and 5% defibrinated horse blood (TCS Biosciences Limited, Botolph Claydon, Buckingham, UK (HB034)). These plates were incubated at 37 °C for 48 h under anaerobic conditions as previously described [15].

DNA was extracted from isolated colonies with the typical *C. difficile* morphology (irregular ground-glass colonies) using the DNeasy blood & tissue kit (QIAGEN GmbH, Hilden, Germany (69504)) and was confirmed as *C. difficile* by testing for the *tpi* gene using PCR [39]. Briefly, 12.5 µL of Mastermix (QIAGEN Ltd., Manchester, UK (206143)), 0.625 µL of each primer (0.5 µM), 9.25 µL of nuclease-free water (Invitrogen, Biosciences Ltd., Dún Laoghaire, Dublin, Ireland (LSKNF0500)) and 2 µL of template were mixed together and amplified using a Veriti 96-Well Thermal Cycler (Applied Biosystems, Warrington, Cheshire, UK). The PCR cycle started with denaturation for 3 min at 95 °C, followed by 40 cycles of 30 s at 95 °C, 30 s at 55 °C and 30 s at 72 °C, and a final extension of 30 s at 72 °C. Electrophoresis in a 2% agarose gel stained with SYBR™ Safe DNA Gel Stain (Biosciences Ltd., Dún Laoghaire, Dublin, Ireland (S33102)) was then used to confirm the *tpi* PCR fragment generated (230-bp) under UV light. *C. difficile* RT078, obtained from a clinical isolate at St. James’s Hospital (Dublin, Ireland) and supplied by Prof. Thomas Rogers from Trinity College Dublin, and *C. sporogenes*, available in the Teagasc Ashtown Food Research Centre collection, were used as the positive and negative controls, respectively.

### 2.3. Characterization of Toxin and Accessory Genes

The confirmed *C. difficile* isolates obtained were further characterised for toxin (*tcdA*, *tcdB*, *cdtA* and *cdtB*) and accessory genes (*tcdC* and *tcdR*) by conventional PCR. Primer sequences, concentration and amplification protocols for toxin A (*tcdA*), toxin B (*tcdB*), toxin CDT (*cdtA* and *cdtB*) and accessory genes (*tcdC* and *tcdR*) were as described by Marcos et al. [24]. The total PCR mixture for every reaction had a final volume of 25 µL with the corresponding volume for each primer depending on its concentration, 12.5 µL of Mastermix (QIAGEN), 2 µL of template and nuclease-free water to reach the total volume. As previously described, a 2% agarose gel stained with SYBR™ Safe DNA Gel Stain (Biosciences) was carried out to separate the PCR fragments: 369-bp for *tcdA*, 160-bp for *tcdB*, 375-bp for *cdtA*, 510-bp for *cdtB*, 718 bp for *tcdC* and 300 bp for *tcdR*. *C. difficile* RT078 and *C. sporogenes* were the corresponding positive and negative controls, respectively.

### 2.4. Antimicrobial Susceptibility Testing

The susceptibility of isolates to a range of relevant antibiotics (vancomycin, erythromycin, metronidazole, clindamycin, moxifloxacin and rifampicin) was tested using the E-test strips (bioMérieux, Marcy-l’Étoile, France), following the protocol described by the manufacturer. The aforementioned antibiotics were selected for testing due to their common use in CDI treatment [40,41,42,43,44].

Isolates were cultured in Mueller–Hinton broth (Oxoid, Basingstoke, Hampshire, UK (CM0405)) at 37 °C for 24 h or until an OD_600 nm_ = 0.5 (10^8^ CFU/mL) was achieved, measured using a DeNovix DS-C spectrophotometer (DeNovix Inc., Wilmington, NC, USA). A sterile swab (Sparks Lab Supplies, Dublin, Ireland (SW001)) was dipped into the broth solution and then spread on Brucella agar plates with vitamin K and haemin (Sigma-Aldrich, Gillingham, UK (B2926-500G)) and 5% defibrinated horse blood (TCS Biosciences Limited, Botolph Claydon, Buckingham, UK (HB034)). Plates were allowed to dry for 15 min before the E-test strip was placed on top of the inoculated agar with a sterile forceps and incubated for 48 h at 37 °C in anaerobic conditions as previously described. After incubation, MIC values were read using the scale (µg/mL) provided by the manufacturer. Values obtained for each antibiotic were compared to the breakpoint values described in EUCAST [45] and classified as susceptible or resistant according to the criteria.

### 2.5. PCR-Ribotyping

Ribotype testing of all isolates obtained was carried out following the PCR ribotyping protocol by ECDC [46], with minor changes. Amplification of the 16S and 23S rRNA genes was undertaken to identify the specific intergenic spacer region (ISR). Primers for the amplification of the *C. difficile* 16S (forward primer) and 23S (reverse primer) rRNA genes were as described by Bidet et al. [47] (5′ GCTGGATCACCTCCTTT CTAAG (6FAM-16S) and 5′ TGACCAGTTAAAAAGG TTTGATAGATT (23S)). The PCR required 12.5 µL of HotStarTaq Mastermix (QIAGEN Ltd., Manchester, UK), 0.25 µL of each primer (10 μM), 10 µL of RNase-free water (QIAGEN Ltd., Manchester, UK) and 2 µL of the template. The mixture was added to a MicroAmp^TM^ Optical 96-well reaction plate (Applied Biosystems, Warrington, Cheshire, UK) before being sealed and inserted in a Veriti 96-Well Thermal Cycler (Applied Biosystems, Warrington, Cheshire, UK). Amplification consisted of a denaturation of 15 min at 95 °C, 30 cycles of 1 min at 94 °C, 1 min at 60 °C and 1 min at 72 °C, and a final extension of 30 min at 72 °C.

After the PCR, denaturation of the generated fragments was necessary before analysis in an automated sequence and fragment analysis system was undertaken. Exactly 2 µL of the PCR product was added to 9.5 µL of Highly Deionized (Hi-Di) Formamide (Applied Biosystems, Warrington, Cheshire, UK) and 0.5 µL of GeneScan 1200 LIZ Size Standard (Applied Biosystems, Warrington, Cheshire, UK). The mix was denatured for 2 min at 95 °C in a thermal cycler, followed by cooling of the plate for 10 min in a fridge before the PCR products were analysed on an ABI 3500 Genetic Analyzer (Applied Biosystems, Warrington, Cheshire, UK) with default settings for POP7 and 50 cm capillary length. The raw data files (*fsa files) from the ABI 3500 Genetic Analyzer were uploaded to the free-to-use website WEBRIBO (https://webribo.ages.at/; accessed on 7 July 2022) to compare our isolates with existing ribotypes stored on the database [13].

### 2.6. Whole Genome Sequencing (WGS)

The *C. difficile* isolates (n = 8) obtained from 2 spinach leaves and 6 soil samples were selected for WGS in order to identify genetic determinants of virulence and resistance. WGS was performed in Teagasc Food Research Centre Moorepark by Dr. Fiona Crispie and Dr. Gaston Allende. DNA libraries were prepared with the Illumina DNA preparation kit as per the manufacturer’s instructions. This was followed by 2 × 150 bp sequencing on the NextSeq 2000 with P2 reagents. Nucleotide sequence data reported are available in the GenBank database under the accession number PRJNA884639.

After quality analysis (FastQC v0.11.8) [48], sequences were trimmed using Trimmomatic (v.0.38) [49]. Paired sequences were subjected to taxonomic annotation using Kraken2 (v.2.1.1) [50] to initially identify the isolates as *C. difficile*, and fragments were de novo assembled using SPAdes (v.3.15.3) [51]. After the contigs’ quality was analysed (Quast v.5.1.0) [52], functional gene annotation was carried out via Prokka (v.1.14) [53]. Further taxonomic analysis was carried out with the contigs using KmerFinder (v.3.2) [54,55,56]. The presence of putative toxin genes (*tcdA*, *tcdB*, *cdtA*, *cdtB*) and plasmids was analysed using ABRicate (v.1.0.1) (PlasmidFinder and Virulence Factor Database (Vfdb)) [57,58,59]. The presence of antimicrobial resistance genes was determined using the ResFinder function in ABRicate and Resistance Gene Identifier (RGI) and CARD [60,61]. Allelic profile analysis was carried out via MLST (v.2.18.0) [62] and uploaded to the open-access online platform PubMLST (http://pubmlst.org, accessed on 23 June 2022) to determine the sequence type (ST) and clade. Single nucleotide polymorphism (SNP) analysis was performed using Snippy (v.4.3.6), using the genome of the *C. difficile* 630 strain as a reference [63]. The pangenome analysis of the *C. difficile* isolates was performed using anvi’o (v7.1) as per the workflow for microbial pangenomics (https://merenlab.org/2016/11/08/pangenomics-v2/; accessed on 27 September 2022) [64]. For this purpose, assembled scaffold.fasta files were transformed into anvi’o contig databases using the ‘anvi-gen-contigs-database’ program. Then, genes in scaffolds were identified via Prodigal to screen for open reading frames and were annotated using the NCBI’s Clusters of Orthologous Groups database (‘anvi-run-ncbi-cogs’ program) [65,66] against four provided HMM profiles of anvi’o using hidden Markov models (‘anvi-run-hmms’ program). The pangenome was computed by calculating the amino acid sequence similarities and comparing them across all genomes via NCBI blastp. In addition, weak matches between amino acid sequences were eliminated using minbit heuristics of 0.5 [67] and clusters were identified using the MCL algorithm [68] (‘anvi-pan-genome’ program). The pangenome was visualized and edited using the anvi’o interactive interface and the program ‘anvi-display-pan’, and the arrangement and quality of figures was improved using Affinity Designer (v1.8.5.703, Serif). A phylogenetic tree was created using the NDtree 1.2 program available in the Center for Genomic Epidemiology [69,70,71].

## 3. Results

### 3.1. Characterisation of the C. difficile Isolates Using Conventional PCR, E-Test and PCR-Ribotyping

**Figure 2:**
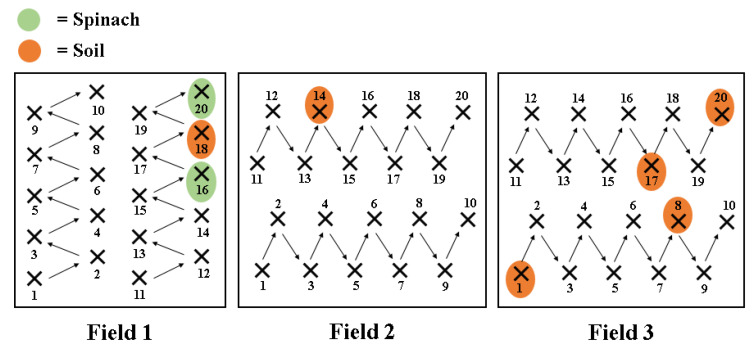
Location of the *C. difficile* positive samples in the 3 fields tested.

A total of 8 (two spinach and six soil) out of 120 samples analysed (6.7%) were confirmed as *C. difficile* positive (Figure 2). In Field 1, two spinach and one soil samples were positive, while one positive soil sample was obtained in Field 2 (Figure 2). Half of the positive samples (four soils) were obtained in Field 3.

The presence or absence of toxin and accessory genes in the *C. difficile* isolates is shown in Table 1. Toxin-encoding genes were detected by PCR in seven of the eight isolates. The *tcdA* and *tcdB* genes were detected in five out of the six soil isolates, while *tcdB*, *cdtA* and *cdtB* were present in the two spinach isolates. Accessory genes (*tcdC* and *tcdR*) were detected in all isolates except in isolate 7, which also lacked the PaLoc.

The resistance or susceptibility of the *C. difficile* isolates to the six antibiotics tested and their corresponding ribotype is also shown in Table 1. All isolates, except for one spinach isolate (ribotype 078) from Field 1, were resistant to at least one antibiotic. Resistance to rifampicin was observed in 5/8 of the isolates, followed by erythromycin (3/8), vancomycin (2/8), clindamycin and moxifloxacin (1/8 each). All isolates were susceptible to metronidazole. Two soil isolates from Fields 2 (RT003) and 3 (RT050) were resistant to the same three antibiotics (erythromycin, vancomycin and rifampicin). The rest of the isolates presented varied resistance and ribotype patterns.

**Table 1:** Ribotype (PCR-ribotyping) and antibiotic susceptibility testing profile using E-test against erythromycin (ERY), metronidazole (MET), clindamycin (CLIN), moxifloxacin (MOX), vancomycin (VAN) and rifampicin (RIF) (S = susceptible; R = resistant) and virulence gene (*tcdA*, *tcdB*, *cdtA*, *cdtB*, *tcdC*, *tcdR*) of the *C. difficile* strains isolated from spinach fields.

Collection Field	Sample Type	ID ^1^	RT ^2^	Antibiotic Resistance Profile (MIC)						Toxin Genes ^3^				Accessory Genes ^3^	
				ERY	MET	CLIN	MOX	VAN	RIF	*tcdA*	*tcdB*	*cdtA*	*cdtB*	*tcdC*	*tcdR*
Field 1	Soil	1	614	S (0.25)	S (0.125)	S (8)	S (1)	S (0.75)	R (0.25)	+	+	−	−	+	+
	Spinach	2	078	S (0.25)	S (<0.016)	S (<0.016)	S (0.38)	S (1.5)	S (<0.002)	−	+	+	+	+	+
		3	126	**R (>256)**	S (0.094)	S (4)	S (0.75)	S (1.5)	S (0.003)	−	+	+	+	+	+
Field 2	Soil	4	003	**R (8)**	S (0.25)	S (1.5)	S (1)	**R (>256)**	**R (0.125)**	+	+	−	−	+	+
Field 3	Soil	5	087	S (0.25)	S (0.125)	S (<0.016)	**R (4)**	S (1.5)	**R (0.047)**	+	+	−	−	+	+
	Soil	6	050	**R (4)**	S (0.094)	S (1.5)	S (0.5)	**R (3)**	**R (0.25)**	+	+	−	−	+	+
	Soil	7	511	S (0.75)	S (0.094)	S (3)	S (0.75)	S (1.5)	**R (0.19)**	−	−	−	−	−	−
	Soil	8	014/0	S (<0.016)	S (<0.016)	**R (16)**	S (0.5)	S (1)	S (<0.002)	+	+	−	−	+	+

^1^ ID: number assigned to each isolate. ^2^ RT: Ribotype.^3^ (+/−): Presence (+) or absence (−) of the gene indicated.

### 3.2. Characterisation of the C. difficile Isolates by Whole Genome Sequencing (WGS)

The initial taxonomic annotation using Kraken2 confirmed all isolates were *Clostridioides difficile* (81.68–93.90%) (data not shown). Further species identification using KmerFinder suggested our isolates (1–8) had a high degree of similarity (>95%) with previously sequenced strains including *C. difficile* DSM 27639, M120, DSM 29020, W0023a, CFSAN096664, CFSAN096664, W0003a and DSM 105001, respectively.

The presence of toxin-encoding genes, sequence type (ST) and clade of the spinach (isolates 2 and 3) and soil (isolates 1, 4, 5, 6, 7, 8) isolates are shown in Table 2. The detection of toxin-encoding genes by WGS analysis was in broad agreement with the results obtained by PCR, with the exception of *tcdA* in the spinach isolates. Isolates 2 and 3 (both spinach isolates from Field 1) belonged to clade 5 and ST11. The rest of the isolates (obtained from soil from Fields 1, 2 and 3) belonged to clade 1 and had different STs (19, 12, 46, 16, 79 and 49).

Antibiotic resistance genes detected in the *C. difficile* isolates via ResFinder, Prokka and CARD software tools are shown in Table 3. ResFinder and Prokka analysis detected that isolates 2 and 3 (spinach from Field 1) carried resistance genes for aminoglycosides (*D19aph(3’)-III_1, ant(6)-Ia_1, ant(6)-Ia_2, ant(6)-Ia_3)* and tetracyclines (*tet(M)_10, tet(40)_1, tet(M)_4)* and all soil isolates, except for 7 and 8, had the vancomycin resistance gene *(vanB)*. Genes encoding antibiotic resistance were not detected in isolates 7 and 8 (soil from Field 3) using ABRicate and Prokka. However, according to the analysis of the isolates using Resistance Gene Identifier (RGI) and CARD, all isolates encoded *CDD-1* or *CCD-2* (carbapenem resistance), *qacG* (disinfecting agents and antiseptics resistance) and a mutation in the 23S rRNA that confers the resistance to erythromycin and clindamycin. A spinach isolate (isolate 3) had resistance to nucleoside antibiotics (*SAT-4*), and soil isolates (1, 4, 5, 6, 7 and 8) carried resistance to glycopeptide antibiotics (*vanXY* and *vanR* in the *vanG* cluster). Isolate 5 (soil from Field 3) also encoded the *cdeA* gene, which confers resistance against fluoroquinolones.

A further comparison between the antimicrobial susceptibility profiles found by E-test and CARD is presented in Table 4.

**Table 3:** Antimicrobial resistance (AMR) genes detected in *C. difficile* isolates from spinach (isolates 2 and 3) and soil (isolates 1, 4, 5, 6, 7, 8) samples using ResFinder, Prokka and CARD.

ID ^1^	AMR Using ResFinder and Prokka		AMR Using CARD	
	Gene	Confers Resistance to	Gene	Confers Resistance to
1	*vanB* ^2^	Vancomycin	*vanXY* gene in *vanG* cluster	Vancomycin
			*vanR* gene in *vanG* cluster	
	ND ^3^		*CDD-1*	Beta-lactams
	ND ^3^		*qacG*	Disinfecting agents and antiseptics
	ND ^3^		*Clostridioides difficile* 23S rRNA mutation	Erythromycin and clindamycin
2	*tet(M)_10*	Tetracyclines	*tet(M)*	Tetracyclines
	*ant(6)-Ia_2*	Aminoglycosides	*ant(6)-Ia*	Aminoglycosides
	ND ^3^		*CDD-1*	Beta-lactams
	ND ^3^		*qacG*	Disinfecting agents and antiseptics
	ND ^3^		*Clostridioides difficile* 23S rRNA mutation	Erythromycin and clindamycin
3	*D19aph(3′)-III_1*	Aminoglycosides	*APH(3′)-IIIa*	Aminoglycosides
	*ant(6)-Ia_1*		*aad(6)*	
	*ant(6)-Ia_3*			
	*tet(40)_1*	Tetracyclines	*tet(40)*	Tetracyclines
	*tet(M)_4*			
	ND ^3^		*CDD-1*	Beta-lactams
	ND ^3^		*SAT-4*	Nucleosides
	ND ^3^		*qacG*	Disinfecting agents and antiseptics
	ND ^3^		*Clostridioides difficile* 23S rRNA mutation	Erythromycin and clindamycin
4	*vanB ^2^*	Vancomycin	*vanXY* gene in *vanG* cluster	Vancomycin
			*vanR* gene in *vanG* cluster	
	ND ^3^		*CDD-2*	Beta-lactams
	ND ^3^		*qacG*	Disinfecting agents and antiseptics
	ND ^3^		*Clostridioides difficile* 23S rRNA mutation	Erythromycin and clindamycin
5	*vanB ^2^*	Vancomycin	*vanXY* gene in *vanG* cluster	Vancomycin
			*vanR* gene in *vanG* cluster	
	ND ^3^		*CDD-1*	Beta-lactams
	ND ^3^		*cdeA*	Fluoroquinolones, disinfecting agents and antiseptics
	ND ^3^		*qacG*	Disinfecting agents and antiseptics
	ND ^3^		*Clostridioides difficile* 23S rRNA mutation	Erythromycin and clindamycin
6	*vanB ^2^*	Vancomycin	*vanXY* gene in *vanG* cluster	Vancomycin
			*vanR* gene in *vanG* cluster	
	ND ^3^		*CDD-1*	Beta-lactams
	ND ^3^		*qacG*	Disinfecting agents and antiseptics
	ND ^3^		*Clostridioides difficile* 23S rRNA mutation	Erythromycin and clindamycin
7	ND ^3^		*vanXY* gene in *vanG* cluster	Vancomycin
			*vanR* gene in *vanG* cluster	
	ND ^3^		*CDD-1*	Beta-lactams
	ND ^3^		*qacG*	Disinfecting agents and antiseptics
	ND ^3^		*Clostridioides difficile* 23S rRNA mutation	Erythromycin and clindamycin
8	ND ^3^		*vanXY* gene in *vanG* cluster	Vancomycin
			*vanR* gene in *vanG* cluster	
	ND ^3^		*CDD-1*	Beta-lactams
	ND ^3^		*qacG*	Disinfecting agents and antiseptics
	ND ^3^		*Clostridioides difficile* 23S rRNA mutation	Erythromycin and clindamycin

^1^ ID: number assigned to each isolate. ^2^: The corresponding gene was found only in Prokka analysis. ^3^ ND: Not detected.

In addition, PlasmidFinder detected the genes of the replicon repUS43_1_CDS12738(DOp1) in the spinach isolates 2 and 3 (99.83% and 100% identity), respectively.

**Figure 3:**
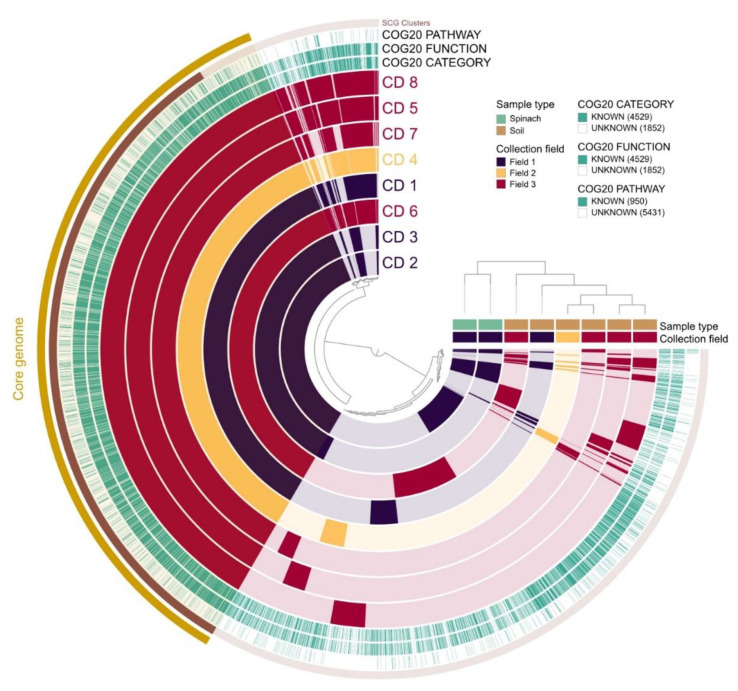
Pangenome analysis for the 8 *C. difficile* isolates from spinach fields. Gene clusters are indicated as opaque coloured elements and ordered based on their presence and absence, using the Euclidean distance and Ward clustering (inner dendrogram). Samples are hierarchically clustered based on the frequency of gene clusters (outer dendrogram). CD ‘number’ = *C. difficile* followed by the isolate ID.

The pangenome analysis carried out for the 8 *C. difficile* isolates is shown in Figure 3. The core genome consisted of 3010 gene clusters and 25,718 genes. In the total pan-genome comparison, 6381 gene clusters were detected with 33,243 genes identified. Two distinct clusters were observed: the first cluster included isolates 2 and 3 (spinach), while the second cluster included the soil isolates (1, 4–8).

**Figure 4:**
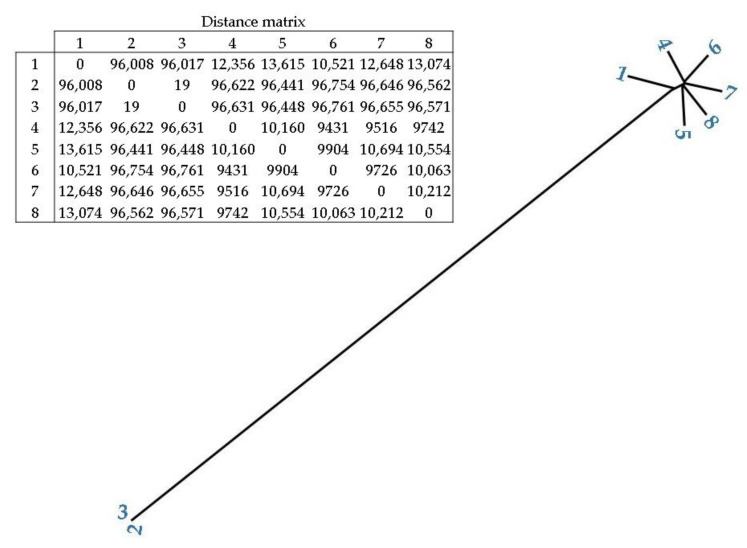
Phylogenetic tree of the *C. difficile* spinach (2 and 3) and soil isolates (1, 4–8) with the distances between isolates presented as a distance matrix.

In addition, Figure 4 shows the phylogenetic relationship and distance between the *C. difficile* isolates. Isolates 2 and 3 (spinach isolates) had the closest distance (19), followed by soil isolates 4 and 6 (9431), and 4 and 7 (9516). Spinach isolates (2 and 3) presented a high distance from all of the soil isolates (>96,008).

## 4. Discussion

### 4.1. Prevalence of C. difficile

*C. difficile* was detected in 3.3% of spinach and 10% of soil samples. This was not unexpected as these bacteria have been previously isolated in compost (22.5%) [37], garden soil (79%), manure (83%) [26] and from soil samples in rural and urban areas (36.7%) [8]. Eckert et al. [31] reported a similar prevalence of *C. difficile* in salads purchased from retail stores in France (3.3%). A slightly lower prevalence was reported in lettuce (1.9%) in an Italian study by Primavilla et al. [34], but higher levels have also been found in salad leaves and ready-to-eat salads in Iran (5.6%) [36], in Ireland (6%) [24] and in Scotland (7.5%) [35]. Root vegetables are more frequently contaminated with *C. difficile* (10–75%) than leafy greens [31,33,35,74], most likely due to soil residues [74,75].

### 4.2. Ribotypes of C. difficile

In our study, ribotypes 078 and 126 were detected on spinach leaves while RT614, 003, 087, 050, 511 and 014/0 were found in the soil. The majority of these ribotypes have been previously linked to animal, food and human sources.

Ribotype 078 is frequently reported in farm animals such as cattle [76,77,78], poultry [79,80] and pigs [81,82,83], with no distinguishable differences between human and porcine RT078 isolates [84,85]. It has also been found in food products derived from animals purchased at retail such as ground pork, turkey [86] and beef [87]. In vegetables, it was detected in ginger and carrots from Canada [33] and in potatoes from Ireland and Italy [28]. In humans, hypervirulent *C. difficile* RT078 accounted for 17% of ribotyped isolates in 2021 in Ireland [88], with similar data reported for Germany (16.7%) by Rabold et al. [89]. Interestingly, the RT078 strain isolated in our study was identified as similar to *C. difficile* strain M120 by WGS, a strain that has been associated with clinical infection in the UK and Ireland [19], as well as in calves in Canada [90] and the pig farm environment in Spain [91].

Ribotype 126 has been described in cattle [76,92,93] and pigs [94,95]. It may not be a coincidence that both ribotypes RT078 and RT126, which are frequently reported in cattle, were detected in the spinach isolates from Field 1 that was located adjacent to fields with bovine animals. Apart from farm animals, Agnoletti et al. [96] and Troiano et al. [97] isolated RT126 from shellfish. Tkalec et al. [28] confirmed the presence of ribotype 126 in potatoes from France, Spain, Austria and Romania, and Primavilla et al. [34] found this ribotype in lettuce served in Italian hospitals. In Spain, RT126 was one the most common ribotypes among clinical isolates [98], while in Portugal, it accounted for 3.8% of the CDI cases [99]. Recently, Azimirad et al. [100] identified RT126 as the predominant ribotype (11.2%) in hospital patients in Tehran. Hypervirulent RT126 was observed in clinical isolates in Taiwan by Hung et al. [101], in Kuwait by Jamal and Rotimi [102] and in Australia by Knight et al. [82]. The ribotype 126 detected in spinach leaves in our study was similar to *C. difficile* strain DSM 29020, which has been reported in hospital patients in Korea and China [103,104].

Of our soil isolates, ribotype 003 was previously reported in poultry faeces (1%) [105] and meat (28.6%) [106], calves [76] and horses (5.6%) [107]. Indeed, the close proximity of the field where it was isolated to a stud farm might highlight the potential for its zoonotic transfer. Regarding its link to humans, in Germany, RT003 accounted for 11.1% of CDI cases in 2012–2013 [89], and strain W0023a, which is similar to our isolate by WGS, has been detected in clinical isolates from Korea and the USA [103,108].

RT087, the ribotype of another of our soil isolates, was identified in poultry meat by de Boer et al. [106] and Tkalec et al. [109] (14.3 and 20%, respectively). The latter authors also reported that ribotype 087 was more common in clinical isolates than RT078 in a Slovenian study.

To the best of our knowledge, this is the first time RT050 has been reported in soil samples, although it has been reported in pigs (12%) [110] and was recently associated with hospitalized adult patients in Mexico [111] and in a CDI outbreak in the intensive care unit of the Amsterdam University Medical Centre [112]. Furthermore, both RT087 and RT050 were similar to *C. difficile* CFSAN096664, which was previously isolated from hospital patients in the USA [113].

Ribotype 014, previously described in retail ground beef (8.3%) [114], was found in soil in Field 3 that was located next to a dairy farm. A variant of this ribotype, RT014/020, was the predominant ribotype among soil isolates obtained from domestic gardens in Australia by Shivaperumal et al. [26]. In humans, a study from Germany noted RT014/0 as the most prevalent ribotype among CDI patients (22.2%) [89], and our WGS analysis suggested that this isolate was similar to *C. difficile* DSM 105001, which has been reported before in US hospital patients [115].

Isolates of RT614 have not been previously reported in soil but have been found in clinical samples in Portugal [116]. This isolate was similar to DSM 27639, which has been reported in German hospital patients [117].

Our remaining soil isolate was ribotype 511 and has not been associated with animal, food or human sources. However, due to the ribotype’s lack of toxin genes, it may be carried asymptomatically [118] or be associated with hospital-acquired diarrhoea [119]. WGS analysis suggested that RT511 was most closely related to strain *C. difficile* W0003a, previously observed in clinical isolates in Korea [103].

### 4.3. Toxigenicity of the C. difficile Isolates

The *C. difficile* population consists of six distinct phylogenetic clades labelled 1, 2, 3, 4, 5, and C-I [120,121]. Most genotypes within clades 1–3 produce both toxins A and B and, in addition to these, strains from clades 2, 3, and 5 have genes encoding the binary toxin, CDT. Clade 5 includes ribotype 078, which is typically associated with community-acquired cases and has a higher rate of complications and mortality in humans [3,110,122]. In the present study, spinach isolates (isolates 2 and 3) both belonged to this highly toxigenic clade 5 (ST11) and contained the binary toxin CDT, characteristic of hypervirulent strains [10], apart from toxins A and B. Interestingly, while conventional PCR yielded a negative result for the *tcdA* gene in the spinach isolates, this gene was detected using WGS. Thus, SNP analysis was carried out to search for possible mutations in the primer region used to amplify *tcdA* that could have affected the PCR analysis and might explain the negative result. However, no SNPs were detected in the primer or promoter region. The use of a positive *tcdA* control (*C. difficile* RT078) and a negative control belonging to *C. sporogenes* in the conventional PCR analysis ruled out issues with the PCR reaction (data not shown), and thus, further research is required to explain this apparent anomaly.

All soil isolates (isolates 1, 4–8) belonged to clade 1 and presented the toxigenic sequence types and ribotypes ST19/RT614, ST12/RT003, ST46/RT087, ST16/RT050, and ST49/RT014/0 and one non-toxigenic ST79/RT511 (isolate 7). Clade 1 comprises more than 200 toxigenic and non-toxigenic sequence types, with most toxigenic strains encoding genes for toxin A and B production, as we observed in the majority of the soil isolates. Moreover, a few STs (ST2, ST8 and ST17) from clade 1 have been associated with CDI cases worldwide [82].

Pangenome and phylogenetic analysis showed the existence of two clusters among our samples. The first cluster included the spinach samples belonging to clade 5 and the second cluster comprised the soil samples that belonged to clade 1. Thus, the soil samples were closely related to each other but were phylogenetically distinct from the spinach isolates (who were, in turn, closely related). However, due to the limited sample size of this study, further studies would need to investigate this potential relationship considering a larger number of isolates.

### 4.4. Antibiotic Susceptibility of the C. difficile Isolates

An interesting finding in our study was the resistance differences observed in antibiotics tested phenotypically and genotypically, supporting previous similar observations in *C. difficile* by Muñoz et al. [123].

Phenotypic resistance to erythromycin (RT126) was observed in a spinach isolate, while soil isolates were resistant to erythromycin, vancomycin and rifampicin (RT003 and RT050), moxifloxacin and rifampicin (RT087), and rifampicin (RT614 and RT511) and clindamycin (RT014/0). While phenotypic analysis was carried out for a limited range of antibiotics (erythromycin, metronidazole, moxifloxacin, clindamycin, vancomycin and rifampicin), genotypic analysis suggested the existence of more resistance determinants. In spinach isolates (RT078 and RT126), resistance genes against tetracycline (*tet(M)* and *tet(40)*), aminoglycosides (*APH(3′)* and *ANT(6)*), beta-lactam (*CDD-1* and *CDD-2*), disinfecting agents and antiseptics (*qacG*), nucleoside (*SAT-4*) and clindamycin antibiotics were detected, while in soil isolates (RT614, 003, 087, 050, 511 and 014/0), resistance genes against vancomycin (*vanR* and *vanXY*), fluoroquinolones (*cdeA*), beta-lactam, disinfecting agents and antiseptics, erythromycin and clindamycin antibiotics were detected. Interestingly, with the exception of erythromycin resistance in isolates 3, 4 and 6, in clindamycin resistance in isolate 8 and vancomycin resistance in isolates 4 and 6, there was little agreement between the phenotypic and genotypic assessment of AMR.

In spinach isolates, similar susceptibilities to those observed in this study in RT078 against all antibiotics tested were described previously in isolates from humans by Álvarez-Pérez et al. [98], rabbits by Drigo et al. [124] and pigs by Zhang et al. [83]. Freeman et al. [14] observed vancomycin resistance in RT078 isolates while we observed susceptibility, although the lack of vancomycin resistance genes we reported in this ribotype would explain our results. In addition, Zhang et al. [83] found resistance to tetracycline in RT078 isolates from pigs in China.

In RT126, phenotypic resistance to erythromycin had been previously reported by Álvarez-Pérez et al. [98] and can be explained by the existence of a mutation in the target site of *C. difficile* 23S rRNA that confers resistance against the antibiotic. Furthermore, the gene SAT-4, which encodes nucleoside antibiotic resistance and was identified in RT126 in our study, was detected in *Campylobacter coli* and *Enterococcus faecium* by other authors [125,126].

Apart from this, both spinach isolates (RT078 and RT126) contained components of the replicon repUS43_1_CDS12738(DOp1), which has been linked with resistance against tetracycline, methicillin and penicillin in methicillin-resistant *Staphylococcus aureus* (MRSA) LA-M*RSA SA0385* [127].

In all soil isolates (RT614, RT003, RT087, RT050, RT511, RT014/0), vancomycin resistance genes (*vanXY* and *vanR*) were detected, even though phenotypic resistance was only observed in isolates of RT003 and RT050, contrary to the susceptibility reported by Kecerova et al. [107] in RT003. These results support the findings of Suzuki et al. [128] in *C. difficile* RT027 strains that carried *vanRG* and *vanG* genes but were phenotypically susceptible to vancomycin. Therefore, the presence of vancomycin resistance genes in *C. difficile* does not always result in their expression in vitro.

Phenotypic resistance to clindamycin in RT014/0, previously reported in human and pig isolates in Australia [129,130] and to erythromycin in RT003 and RT050, is probably due to the mutation detected in the antibiotic target site of *C. difficile* 23S rRNA, which confers resistance to both antibiotics. The resistance of *C. difficile* strains to erythromycin and clindamycin has been widely reported in human isolates and treatment with clindamycin is a risk factor for developing CDI [18,131].

Additionally, the in vitro resistance to moxifloxacin in RT087, in contrast to the results reported by Freeman et al. [14] for this ribotype, could be explained by the presence of the *cdeA* gene. This gene confers resistance against fluoroquinolones in *E. coli* and has been previously detected in *C. difficile* [132].

To the best of our knowledge, no previous authors have described antimicrobial resistance patterns for RT614 and RT511, possibly due to a lower frequency of reports in humans, even though they both showed phenotypic resistance to rifampicin in our study and have been associated with an increased severity of CDI [133].

The *CDD-1* and *CDD-2* genes, detected in all of the isolates analysed in this study, confer a high resistance against a broad range of beta-lactams [134]. While beta-lactam antibiotics were not tested by E-test in this study, these enzymes previously reported in *C. difficile* allow the bacteria to have intrinsic resistance to antibiotics such as penicillin, monobactams and cephalosporins [135].

Interestingly, the *qacG* gene present in every isolate was identified previously in staphylococci isolated from the food industry, conferring resistance to benzalkonium chloride that is a frequently used quaternary ammonium disinfectant [136]. Resistance to quaternary ammonium compounds is plasmid-borne in Gram-positive bacteria, linked to the Small Multi-drug Resistance (SMR) family transporters (*qacC/D* and *qacEΔ1, qacG, qacH* and *qacJ*) [137]. While the *qacG* gene has not been reported in *C. difficile* before, in 2013, it was detected in a *S. aureus* MRSA strain isolated from pork [138]. However, Seier-Petersen et al. [139] described no reduced susceptibility in vitro to biocides in *S. aureus* MRSA strains where the *qacG* gene was present. More recently, the gene was found in the *Staphylococcus* species in Turkey [140] and carbapenem-resistant *A. baumannii* in China [141].

## 5. Conclusions

It was concluded that the prevalence of *C. difficile* was 10% in soil and 3.3% in spinach, both of which were lower than the results reported in other similar studies. Both spinach isolates (toxigenic RT078 and RT126) carried virulence genes (toxins A, B and CDT) and RT126 was phenotypically resistant to erythromycin. Soil isolates included toxigenic ribotypes (003, 014/0, 050, 087 and RT614) and a novel non-toxigenic ribotype (RT511). RT003 and RT050 were resistant to erythromycin, vancomycin and rifampicin, RT087 to moxifloxacin and rifampicin, and RT014/0 to clindamycin. WGS suggested there were inconsistencies between AMR phenotypes and genotypes. Moreover, the soil isolates were closely related but genetically distinct from the spinach strains.

## Figures and Tables

**Table 2:** Virulence profiles of the *C. difficile* isolates from spinach (isolates 2 and 3) and soil (isolates 1, 4, 5, 6, 7, 8) samples after MLST and ABRicate analysis (vdfb).

ID ^1^	ST ^2^	Clade	Toxin Genes ^3^			
			*tcdA*	*tcdB*	*cdtA*	*cdtB*
1	19	1	+	+	−	−
2	11	5	+	+	+	+
3	11	5	+	+	+	+
4	12	1	+	+	−	−
5	46	1	+	+	−	−
6	16	1	+	+	−	−
7	79	1	−	−	−	−
8	49	1	+	+	−	−

^1^ ID: number assigned to each isolate. ^2^ ST: Sequence type. ^3^ (+/−): Presence (+) or absence (−) of the gene indicated.

**Table 4:** Heat map comparing the antimicrobial resistance (AMR) profiles observed by E-test (phenotypic) and CARD (genotypic).

	Genotypic AMR Profile					
ID	23S rRNA Mutation (Erythromycin)	*nimB* Mutation ^1^	23S rRNA Mutation (Clindamycin)	*cdeA*	*vanB*	*RpoB* Mutation ^2^
1						
2						
3						
4						
5						
6						
7						
8						
	Erythromycin	Metronidazole	Clindamycin	Moxifloxacin	Vancomycin	Rifampicin
	Phenotypic AMR profile					
	Only in phenotypic AMR analysis					
	Only in genotypic AMR analysis					
	Found in both genotypic and phenotypic AMR analysis					

^1^ Resistance to metronidazole can be caused by mutations in the gene *nimB* [72]. ^2^ Rifampicin resistance can be due to mutations in the gene *RpoB* [73].

## Data Availability

Nucleotide sequence data reported are available in the GenBank database under the accession number PRJNA884639.
